# Urinary Metabolomic Profiling in Streptozotocin-Induced Diabetic Mice after Treatment with Losartan

**DOI:** 10.3390/ijms21238969

**Published:** 2020-11-26

**Authors:** Jin Seong Hyeon, Youngae Jung, Gayoung Lee, Hunjoo Ha, Geum-Sook Hwang

**Affiliations:** 1 Integrated Metabolomics Research Group, Western Seoul Center, Korea Basic Science Institute, Seoul 03759, Korea; jshyeon@kbsi.re.kr (J.S.H.); jya0819@kbsi.re.kr (Y.J.); 2 Graduate School of Pharmaceutical Sciences, Ewha Womans University, Seoul 03760, Korea; lali7@ewhain.net; 3 Department of Chemistry and Nano Science, Ewha Womans University, Seoul 03760, Korea

**Keywords:** metabolomics, diabetic kidney disease, losartan, NMR

## Abstract

Diabetic kidney disease (DKD) is the leading cause of chronic kidney disease and end-stage kidney disease. Renin–angiotensin system inhibitors such as losartan are the predominant therapeutic options in clinical practice to treat DKD. Therefore, it is necessary to identify DKD-related metabolic profiles that are affected by losartan. To investigate the change in metabolism associated with the development of DKD, we performed global and targeted metabolic profiling using 800 MHz nuclear magnetic resonance spectroscopy of urine samples from streptozotocin-induced diabetic mice (DM) with or without losartan administration. A principal component analysis plot showed that the metabolic pattern in the losartan-treated diabetic mice returned from that in the DM group toward that in the control mice (CM). We found that 33 urinary metabolites were significantly changed in DM compared with CM, and the levels of 16 metabolites among them, namely, glucose, mannose, myo-inositol, pyruvate, fumarate, 2-hydroxyglutarate, isobutyrate, glycine, threonine, dimethylglycine, methyldantoin, isoleucine, leucine, acetylcarnitine, 3-hydroxy-3-methylglutarate, and taurine, shifted closer to the control level in response to losartan treatment. Pathway analysis revealed that these metabolites were associated with branched-chain amino acid degradation; taurine and hypotaurine metabolism; glycine, serine, and threonine metabolism; the tricarboxylic acid cycle; and galactose metabolism. Our results demonstrate that metabolomic analysis is a useful tool for identifying the metabolic pathways related to the development of DKD affected by losartan administration and may contribute to the discovery of new therapeutic agents for DKD.

## 1. Introduction

Diabetic kidney disease (DKD) is a global healthcare burden, and the burgeoning number of patients with DKD implies an urgent need to explore more effective and novel therapeutic targets to control disease progression [1,2]. DKD is treated with inhibitors of the renin–angiotensin system (RAS) as part of the standard treatment regimen [3,4]. RAS inhibitors significantly delay the progression of DKD, although not all patients respond to RAS inhibitors [5,6]. Currently, there are no new therapeutic agents being clinically used. Therefore, it is important to understand the exact pathogenic mechanism of DKD.

Metabolomic analysis is a powerful tool for evaluating systemic metabolic processes and is very useful for investigating disease metabolism [2,7]. As reviewed, several metabolomic studies have reported metabolic changes in the plasma, serum, and urine of patients with DKD, and metabolites such as plasma uremic solutes and urinary tricarboxylic acid (TCA) cycle intermediates are associated with DKD progression [8]. A previous study demonstrated that decreased intrarenal organic toxins are related to the renoprotective effect of fosinopril, which is an angiotensin II converting enzyme (ACE) inhibitor, in renal cortex samples from streptozotocin (STZ)-induced diabetic rats [9]. The effect of losartan belonging to the RAS inhibitor in DKD has been reported to reduce albuminuria and protect the kidney in human and mouse models [3,4,10]. Observational clinical studies observed little effect of losartan on the relationship between the metabolite and glomerular injury in type 2 diabetic patients [11]. The relationship between the renoprotective effect of losartan and changes in metabolic pathways is not known. Therefore, this study is the first to investigate the changes in metabolism caused by administration of RAS inhibitors, such as losartan, in urine samples from a murine model of kidney injury.

Nuclear magnetic resonance (NMR) spectroscopy is one of the analytical techniques commonly used to analyze metabolomes [2]. The advantages of NMR are high reproducibility and minimal sample preparation that was being less prone to artifacts with minimal user interference than other techniques. Several NMR-based metabolomic studies have examined the metabolic change associated with DKD [12,13]. We have previously reported that phase-specific changes in metabolite levels in *db/db* mice during progression of DKD included urinary metabolites associated with glucose and TCA cycle intermediates, branched-chain amino acid (BCAA) and homocysteine-methionine metabolism, and ketone and fatty acid metabolism [14]. Here, we performed global and targeted metabolic profiling of kidney injury in an STZ-induced diabetic mouse model to study DKD and investigated the metabolic pathways affected by the administration of losartan, which is often used experimentally as an RAS inhibitor.

## 2. Results

### 2.1. Characteristics of Experimental Animals

The characteristics of the control mice (CM), STZ-induced diabetic mice (DM), and losartan-treated diabetic mice (LDM) at the time of sacrifice are presented in Table 1. The body weight gain of the DM group was significantly lower than that of the CM group. The blood glucose levels, hemoglobin A1c (HbA1c) levels, and urine volume of DM were significantly increased compared to those of CM. Administration of losartan for 12 weeks did not affect blood glucose or HbA1c levels. It was observed that the urine albumin/creatinine ratio (UACR), which was increased in DM, decreased after losartan administration.

### 2.2. Metabolic Profiling and Pattern Recognition Analysis

**Figure 1:**
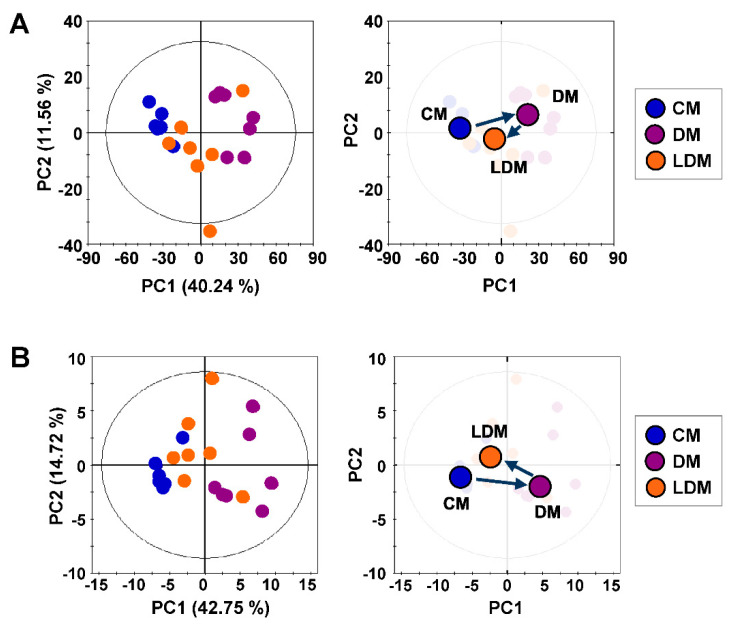
PCA score plots of metabolic profiles of mouse urine. The PCA score plot and trajectory analysis of the PCA score plot (**A**) of global metabolic profiles show perturbation of metabolic patterns in CM (blue dots, *n* = 6), DM (violet dots, *n* = 7), and LDM (orange dots, *n* = 7). The PCA score plot and trajectory analysis of the PCA score plot (**B**) of targeted metabolic profiles were similar to the global metabolic profiles. Circles represent medians of each group. PCA, principal component analysis; CM, control mice; DM, STZ-induced diabetic mice; LDM, losartan-treated diabetic mice.

Proton NMR spectra were obtained for urine samples from CM, DM, and LDM (Figure S1). For global metabolic profiling, principal component analysis (PCA) was performed on the NMR data to examine intrinsic variations among the three experimental groups. The PCA score plot showed that the DM group was markedly separated from CM, with an R^2^ value of 51.8% and a Q^2^ value of 35.1% (Figure 1A). Interestingly, the metabolic pattern of LDM returned from that of DM toward that of CM.

For the targeted metabolite analysis among CM, DM, and LDM, we identified 68 urinary metabolites in the NMR spectra. The PCA score plot of the metabolite concentrations showed good separation between CM and DM (R^2^ = 0.762 and Q^2^ = 0.421) (Figure 1B). Similar to the global profiling result, the metabolic pattern of LDM was directed toward that of CM.

**Figure 2:**
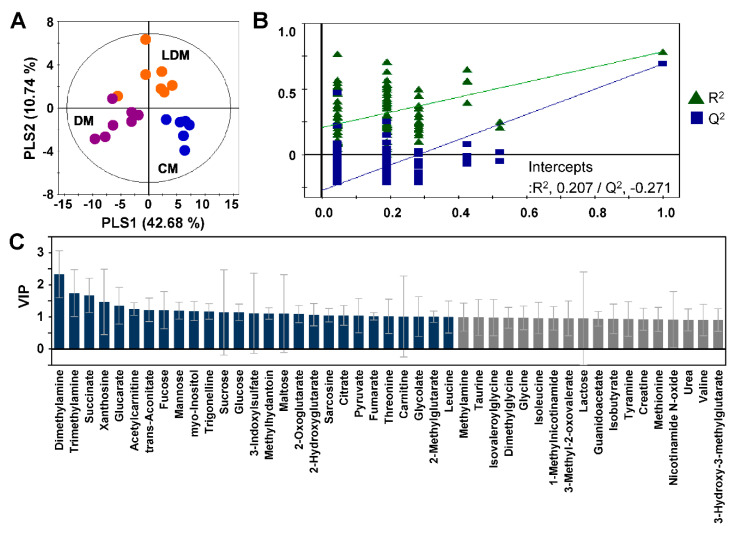
PLS-DA score plot of targeted metabolic profiles of mouse urine. The PLS-DA score plot (**A**) shows perturbation of metabolic patterns in CM (blue dots), DM (violet dots), and LDM (orange dots). Validation plots of urine (**B**) were obtained from 100 permutation tests of the responses to the PLS-DA models. Identified metabolites that have high VIP scores are presented in the bar graph (**C**) in gray (VIP > 0.9) or dark blue (VIP > 1.0). PLS-DA, partial least squares discriminant analysis, CM, control mice; DM, STZ-induced diabetic mice; LDM, losartan-treated diabetic mice.

Partial least squares discriminant analysis (PLS-DA) of the quantified data was performed to observe differences in metabolic patterns among the three experimental groups. The PLS-DA score plots showed a clear separation among the three groups (R^2^ = 0.739 and Q^2^ = 0.594) (Figure 2A). The validity of the PLS-DA model was confirmed by analysis of variance of the cross-validated residuals (CV-ANOVA) (*p* = 2.95 × 10^−5^) and permutation tests (Figure 2B). The variable importance in projection (VIP) scores of the PLS model were used to find the metabolites with significant differences among CM, DM, and LDM from 67 urinary metabolites (excluding creatinine from the 68 identified metabolites). The 45 metabolites with high VIP scores (>0.9) were considered to be important metabolites representing the difference among the three groups (Figure 2C).

### 2.3. Changes in Urinary Metabolites in Response to Losartan Treatment

Among the metabolites displaying VIP scores > 0.9 in the PLS-DA model, 33 metabolites changed significantly between DM and CM (Table S1). The levels of metabolites that are monosaccharides or are associated with the TCA cycle, including glucose, mannose, myo-inositol, pyruvate, 2-oxoglutarate, citrate, fumarate, and trans-aconitate, were significantly increased in DM compared to CM, whereas the levels of succinate decreased significantly. In addition, the levels of amino acid and fatty acid metabolites, including tyramine, glycine, threonine, dimethylglycine, sarcosine, creatine, guanidoacetate, methyldantoin, urea, methionine, isoleucine, leucine, valine, 3-methyl-2-oxovalerate, acetylcarnitine, isovaleroylglycine, 3-hydroxy-3-methylglutarate, and 2-methylglutarate, were significantly increased in DM compared to CM, whereas the levels of taurine was significantly decreased. The levels of glucarate, 2-hydroxyglutarate, isobutyrate, methylamine, and trigonelline were higher in DM than in CM.

**Figure 3:**
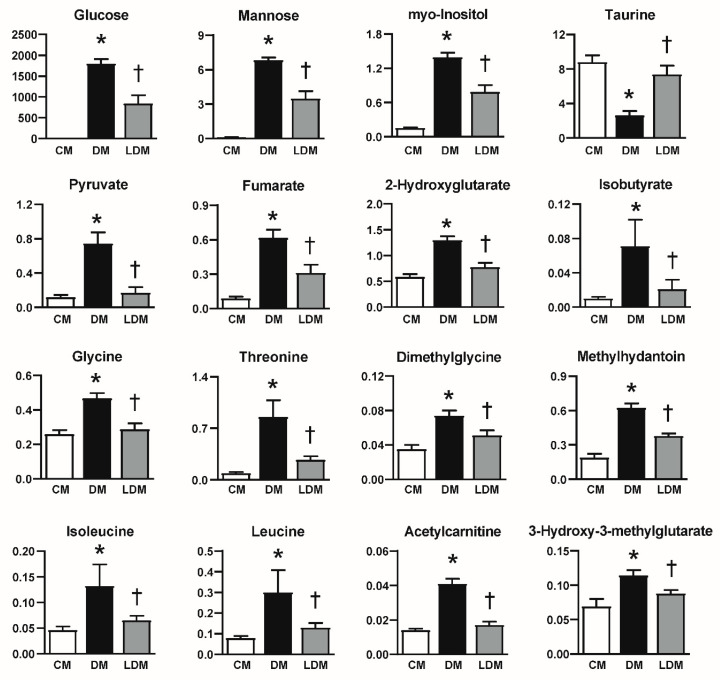
Change of urinary metabolites in LDM compared to DM. Data are presented as the levels of metabolites (metabolite concentration (µM)/urinary creatinine concentration (µM)). * *p* < 0.05, difference between CM and DM. † *p* < 0.05, difference between DM and LDM. CM, control mice; DM, STZ-induced diabetic mice; LDM, losartan-treated diabetic mice.

Of these 33 metabolites, 16 metabolites were observed to change significantly in LDM compared to DM (Figure 3). The levels of glucose, mannose, myo-inositol, pyruvate, fumarate, 2-hydroxyglutarate, isobutyrate, glycine, threonine, dimethylglycine, methyldantoin, isoleucine, leucine, acetylcarnitine, and 3-hydroxy-3-methylglutarate were significantly reduced in LDM compared to DM. On the other hand, taurine levels were significantly higher in LDM than in DM.

### 2.4. Metabolic Pathway Analysis

**Figure 4:**
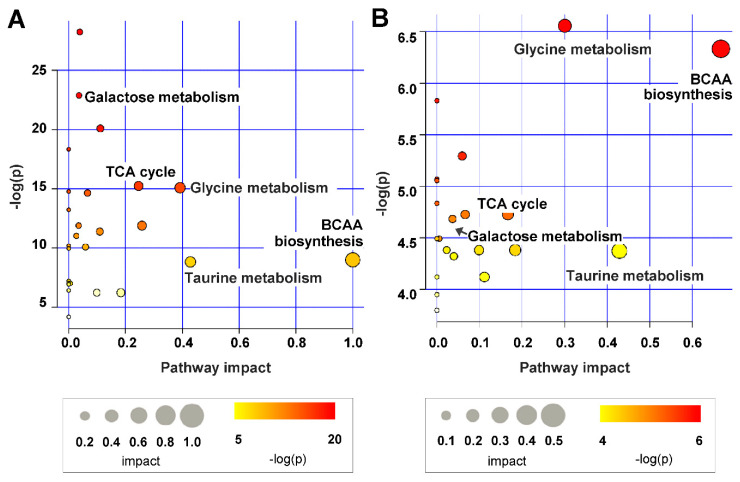
Summary of the analysis of pathways affected by STZ-induced kidney injury (**A**) and losartan treatment (**B**) in urine. The circle size and importance (*x*-axis) represent the pathway impact value calculated pathway topological analysis, and the circle color and direction (*y*-axis) is the -log (*p*-value) obtained from pathway enrichment analysis. The most significant pathways were represented with size and color in the circle from the smallest to the largest and from yellow to red, respectively.

We performed a pathway analysis using the 33 metabolites that changed significantly between DM and CM to reveal the pathways associated with DKD. The results showed that the identified pathways were related to BCAA degradation; taurine and hypotaurine metabolism; glycine, serine, and threonine metabolism; the TCA cycle; and galactose metabolism (Figure 4A). The pathways associated with the 16 metabolites that changed in LDM compared to DM were similar to the metabolic pathways that changed in DKD (Figure 4B).

## 3. Discussion

In this study, we analyzed the change in urinary metabolites associated with losartan administration in an STZ-induced DKD animal model using NMR. Thirty-three metabolites among the 68 identified metabolites were significantly changed between DM and CM. Sixteen of these metabolites showed losartan-mediated recovery from kidney injury in DM toward normal levels. Additionally, we confirmed that the pathways associated with the 16 metabolites that were significantly altered by losartan administration included BCAA degradation; taurine and hypotaurine metabolism; glycine, serine, and threonine metabolism; the TCA cycle; and galactose metabolism.

The previous biological studies showed that losartan effectively prevented kidney injuries such as systemic oxidative stress, albuminuria, tubular injury, podocyte injury, and kidney fibrosis in mouse models of type 1 and type 2 diabetic kidney disease [4,10]. In agreement with the previous studies stating that losartan does not affect blood glucose or HbA1c [4,10], the levels of blood glucose and HbA1c did not change after administration of losartan under our experimental conditions. Considering that human diabetic patients are under the control of hyperglycemia and that activated RAS contributes to increased oxidative stress, inflammation, and fibrosis, our metabolomic results help better understand the renoprotective effect of losartan, as well as give us insight into metabolic pathways targeting DKD.

We found that the levels of leucine and isoleucine in the DM group were markedly increased, but these levels decreased following losartan administration. Previous studies reported that increased BCAA levels in plasma are closely associated with type 1 and 2 diabetes, obesity, and kidney disease [15,16], and increased BCAA levels in urine are observed in the early phases of DKD [14,17]. As mentioned above, blood glucose and HbA1c were not affected by losartan. Thus, changes in urinary BCAA may be related with kidney injury associated with diabetes not hyperglycemia.

Taurine is an intracellular osmolyte associated with taurine and hypotaurine metabolism, and it affects osmoregulation, bile acid conjugation, cell proliferation, viability, and prevention of oxidant-induced tissue injury [18]. It was reported that urinary taurine levels were significantly decreased in patients with chronic kidney disease [19]. Our results showed that the levels of urinary taurine, which decreased in DM, recovered toward control levels after losartan administration. Consistent with our data, the taurine levels in urine were decreased in *db/db* diabetic mice [14], and decreased taurine in the perfused renal cortex of STZ-induced diabetic rats was prevented by fosinopril treatment [9].

We observed increased urinary glycine, threonine, and dimethylglycine levels in DM compared to CM. Glycine is the simplest amino acid, and it passes through the glomerular filtrate barrier due to its small size. It is extensively reabsorbed by the proximal tubules and is present at minimal levels in normal urine. It was reported that elevated glycine levels in the urine are a marker of tubular damage in rats [20]. Dimethylglycine, a glycine derivative, is a well-known inhibitor of betaine-homocysteine methyltransferase and contributes to elevated plasma homocysteine concentrations in patients [21] and animals [22] with renal failure. Another study reported that the levels of urinary dimethylglycine were increased in rats with adenine-induced severe chronic tubulointerstitial nephropathy [23]. Therefore, increased glycine and dimethylglycine levels may suggest tubular injury in DM, which is prevented by losartan. It is noted that threonine, an essential amino acid in animals and humans, was increased in DKD and returned to control level in response to losartan. However, the relationship between urinary threonine and diabetes/kidney disease has not yet been reported.

Pyruvate and fumarate are organic acids related to the TCA cycle. Pyruvate is an important end product of glycolysis by pyruvate kinase M2 (PKM2) and can be converted by the pyruvate dehydrogenase complex to acetyl-CoA, which then enters the TCA cycle. Fumarate is converted to malate by fumarate hydratase (FH) in mitochondria. The mitochondria dysfunction caused by the reduction of peroxisome proliferator-activated receptor-coactivator-1α (PGC1α) [24] and activation of nicotinamide adenine dinucleotide phosphate oxidase 4 (NOX4) [25] in DKD is well known. Moreover, the upregulation of PGC1α by PKM2 activation [26] and elevated FH by the inhibition of NOX4 [27] have a renoprotective effect against DKD. Previous studies have reported that angiotensin II is increased in diabetes [28] and that angiotensin II inhibits PGC1α [29] and activates NOX4 [25] in the kidney as in other tissue [30]. Therefore, it is expected that the use of RAS inhibitors including ACE inhibitors and angiotensin II receptor blockers may inhibit diabetes-induced pyruvate and fumarate upregulation.

Urinary levels of glucose, mannose, and myo-inositol, which are associated with galactose metabolism, were significantly elevated in DM compared to CM and subsequently reduced by approximately 50% following losartan administration. It is well known that glucose in the urine causes osmotic diuresis in diabetes [31]. Interestingly, urine volume was decreased in LDM compared to DM at the presence of hyperglycemia/elevated HbA1c in LDM, and there was no correlation between blood glucose/HbA1c and urine volume in LDM (data not shown). These data suggest that decreased urinary glucose, mannose, and myo-inositol levels in LDM may result from the renoprotective effect of losartan.

In conclusion, NMR-based metabolic profiling was used to detect significant changes in DKD in mice treated with losartan. Although further study is needed to explore the change in metabolism caused by ACE inhibitors or other angiotensin receptor blockers, our findings demonstrate that metabolomic analysis may help to elucidate the DKD-related pathological processes and to discover a new therapeutic agent for DKD.

## 4. Material and Methods

### 4.1. Animals

Eight-week-old C57BL/6 mice were purchased from Japan SLC, Inc. (Hamamatsu, Japan). The mice were divided into the following three groups: (1) control mice (*n* = 6); (2) STZ-induced diabetic mice (*n* = 7); and (3) losartan-treated diabetic mice (*n* = 7) (Figure S2). Losartan was administered starting at 2 days after the final injection of STZ when hyperglycemia was confirmed. Diabetes was induced by intraperitoneal injection of 50 mg/kg STZ for 5 days, with age-matched control mice injected with an equivalent volume of sodium citrate buffer (100 mM sodium citrate and 100 mM citric acid; pH 4.5). The mice were housed in a room maintained at 22 ± 2 °C under a 12 h/12 h light/dark cycle. Losartan at 1.5 mg/kg/day was orally administered to STZ-induced diabetic mice for 12 weeks. The dose of losartan was determined based on our preliminary data [14]. Before sacrifice, blood and 24-h urine samples were collected from nonfasting mice housed in metabolic cages. Urine samples were stored at −80 °C until NMR analysis. All animal experiments were approved by the Institutional Animal Care and Use Committee of Ewha Womans University (IACUC No. 14–051, Permit date: 01-07-2014).

### 4.2. Biochemical Analysis

Blood glucose and HbA1c levels were measured with a glucometer (OneTouch Ultra; Johnson & Johnson, Milpitas, CA, USA) and with a DCA2000 HbA1c Reagent Kit (Siemens Healthcare Diagnostics, Tarrytown, NY, USA), respectively. Urinary albumin was measured using a competitive enzyme-linked immunosorbent assay (ALPCO, Westlake, OH, USA). Urine spectra were used to determine the urine creatinine concentration, and urine albumin levels were corrected for urine creatinine and presented as the UACR.

### 4.3. ^1^H-NMR Samples and Experiments

Prior to NMR analysis, frozen urine samples were thawed at room temperature and centrifuged at 12,000 rpm for 10 min at 4 °C to acquire the supernatant, 100 μL of which was mixed with 500 μL of 1 mM sodium azide in 0.2 M sodium phosphate buffer (pH 7.0). After adjusting the pH to 7.0 ± 0.1, a 540 μL sample was mixed with 60 μL of 2 mM 3-(trimethylsilyl) propionic 2,2,3,3-acid (TSP) in D_2_O.

One-dimensional (1D) ^1^H-NMR spectra were acquired with an Ascend 800 MHz AVANCE III HD Bruker spectrometer (Bruker BioSpin AG, Fällanden, Switzerland) using a triple-resonance 5 mm CPTIC cryogenic probe. Bruker standard 1D nuclear Overhauser enhancement spectroscopy (NOESY) preset (noesypr1d) pulse sequences were used with the following parameters: short delay, 12.3 µs; *n*, 128; dummy scans, 16; *Acq*, 2.0 s; and mixing time, 10 ms. Free induction decay information was acquired at 65,536 data points with a spectral width of 20 ppm. Signal assignment for representative samples was facilitated by the acquisition of 2D correlation spectroscopy (COSY) and heteronuclear single quantum correlation (HSQC) data.

### 4.4. NMR Spectral Preprocessing and Statistical Analysis

NMR data were processed using TopSpin software (v.3.1; Bruker BioSpin AG). All spectra were manually phase corrected and baseline corrected, and the urine spectra were calibrated using TSP. Global metabolic profiling allows rapid assessment of the metabolic patterns among groups by combining spectral data and multivariate statistical analyses. For global metabolic profiling, the processed NMR spectra were segmented with equal widths into 0.005 ppm bins using Chenomx software (v.7.1; Chenomx, Edmonton, AB, Canada). Binning data were normalized to the creatinine concentration of each spectrum in which the water-peak region (4.60–5.05 ppm) was removed. The data files were imported into MATLAB (R2008a; Mathworks, Inc., Natick, MA, USA), and all spectra were aligned using the correlation-optimized warping algorithm [31].

Targeted metabolic profiling was used to compare the concentration of quantified metabolites among groups. For targeted metabolic profiling, NMR spectra were imported into Chenomx for identification and quantification. The 800 MHz Chenomx library, as well as COSY and HSQC spectra, was used to identify individual compounds. The quantified results were normalized according to urinary creatinine levels (metabolite μM/creatinine μM). The assignment of ambiguous peaks due to peak overlap was confirmed by spiking with standard compounds.

### 4.5. Statistical Analysis

The resulting datasets were unit variance scaled, which was performed for multivariate statistical analyses using SIMCA-P+ (v.12.0; Umetrics, Umea, Sweden). PCA was used as an unsupervised pattern recognition method for analyzing samples without group information to reveal outliers and obtain an overview of intrinsic variations in the dataset. PLS-DA, a supervised classification method, was used to maximize class discrimination and identify the important metabolites. The Q^2^ (predicted variation, “goodness of predictability”) and R^2^ (explained variation, “goodness of fit”) parameters were used to evaluate the models. Additionally, the significance of PLS-DA models was validated by CV-ANOVA and permutation tests using 100 random permutations. Statistical analyses were performed using SPSS (v.21.0; IBM Corp., Armonk, NY, USA). One-way analysis of variance (ANOVA) after conversion into ranking variables was used to detect differences in metabolites among the three groups. Tukey’s post hoc test for multiple comparisons was used after one-way ANOVA. *p* < 0.05 was considered statistically significant. MetabolAnalyst 4.0 (http://www.metaboanalyst.ca) was used to reveal the metabolic pathways related to STZ-induced kidney injury and losartan administration [32].

## Figures and Tables

**Table 1:** Baseline characteristics of control mice (CM), STZ-induced diabetic mice (DM), and losartan-treated diabetic mice (LDM).

	CM (*n* = 6)	DM (*n* = 7)	LDM (*n* = 7)
Body weight (g)	26.6 (26.0, 26.9)	22.6 (21.3, 23.1) ***	25.3 (23.3, 25.9)
Blood glucose (mmol/L)	192.5 (181.0, 223.0)	519.0 (512.5, 543.0) ***	509.0 (469.0, 522.0)
HbA1c (%)	4.2 (4.1, 4.2)	8.6 (8.4, 9.2) ***	7.2 (6.6, 8.2)
Urine volume (mL/day)	0.9 (0.6, 1.3)	19.6 (17.6, 26.4) ***	6.4 (4.3, 9.6) ^†^
UACR (mg/mmol)	22.1 (20.3, 26.7)	102.2 (99.1, 112.9) **	36.9 (35.2, 49.6) ^††^

The results are presented as the median (25th, 75th percentiles). * *p* < 0.05, ** *p* < 0.01, and *** *p* < 0.001 for the difference between CM and DM. ^†^ *p* < 0.05, ^††^ *p* < 0.01, and ^†††^ *p* < 0.001 for the difference between DM and LDM. HbA1c, hemoglobin A1c; UACR, urinary albumin/creatinine ratio; STZ, streptozotocin.

## Supplementary Materials

The Supplementary Materials are available online at https://www.mdpi.com/1422-0067/21/23/8969/s1.
