# Supplementary material for: Urinary Metabolomic Profiling in Streptozotocin-Induced Diabetic Mice after Treatment with Losartan
Source: Int J Mol Sci. 2020 Nov 26;21(23):8969. doi: 10.3390/ijms21238969 (PMC7730544; doi:10.3390/ijms21238969)
Supplement: Supplementary file 1 [file ijms-21-08969-s001.pdf]

Urinary metabolomic profiling in streptozotocin-induced diabetic mice after treatment with losartan

Jin Seong Hyeon [1,2], Youngae Jung [1], Gayoung Lee [2], Hunjoo Ha [2,*] and Geum-Sook Hwang [1,3,*]

[1] Integrated Metabolomics Research Group, Western Seoul Center, Korea Basic Science Institute, Seoul, 03759, Republic of Korea; jshyeon@kbsi.re.kr (J.S.H.); jya0819@kbsi.re.kr (Y.J.); gshwang@kbsi.re.kr (G.-S. H.)

[2] Graduate School of Pharmaceutical Sciences, Ewha Womans University, Seoul, 03760, Republic of Korea; lali7@ewhain.net (G.L.); hha@ewha.ac.kr (H.H.)

[3] Department of Chemistry and Nano Science, Ewha Womans University, Seoul, 03760, Republic of Korea.

Correspondence: hha@ewha.ac.kr (H. H.); gshwang@kbsi.re.kr (G.-S. H.)

Table S1. Quantification of urinary metabolites from ^{1}H NMR spectra of CM, DM, and LDM.

**Metabolite**	**CM** (***n*** **= 6)**	**DM** (***n*** **= 7)**	**LDM** (***n*** **= 7)**
Glucose	1.38 (0.98, 2.35)	1796.74 (1639.72, 2067.28) ***	839.76 (670.52, 1095.32) ††
Mannose	0.10 (0.06, 0.11)	6.83 (6.17, 6.96) ***	3.48 (3.16, 4.07) ††
myo-Inositol	0.15 (0.14, 0.18)	1.39 (1.33, 1.57) ***	0.79 (0.70, 1.12) †††
Glucarate	0.10 (0.08, 0.14)	0.30 (0.28, 0.34) ***	0.28 (0.27, 0.29)
Pyruvate	0.12 (0.08, 0.20)	0.74 (0.41, 0.89) **	0.17 (0.11, 0.24) †
2-Oxoglutarate	2.36 (1.53, 2.72)	7.09 (6.70, 7.79) ***	5.37 (4.29, 6.30)
Citrate	8.59 (7.63, 8.92)	18.13 (17.29, 19.27) ***	13.08 (12.02, 18.61)
Succinate	1.18 (1.07, 1.61)	0.81 (0.69, 0.94) *	0.67 (0.58, 0.73)
Fumarate	0.09 (0.06, 0.09)	0.62 (0.47, 0.80) ***	0.31 (0.20, 0.48) †
trans-Aconitate	1.26 (1.12, 1.35)	2.14 (1.93, 2.31) ***	1.87 (1.76, 2.18)
2-Hydroxyglutarate	0.59 (0.50, 0.66)	1.30 (1.03, 1.39) ***	0.77 (0.63, 0.92) ††
Tyramine	0.08 (0.05, 0.18)	0.28 (0.25, 0.30) **	0.22 (0.15, 0.24)
Isobutyrate	0.01 (0.01, 0.01)	0.07 (0.06, 0.16) ***	0.02 (0.02, 0.03) ††
Methylamine	0.51 (0.43, 0.58)	0.87 (0.74, 0.92) **	0.70 (0.68, 0.78)
Glycine	0.26 (0.22, 0.31)	0.47 (0.42, 0.51) **	0.29 (0.27, 0.35) †
Threonine	0.09 (0.06, 0.11)	0.85 (0.52, 1.38) ***	0.27 (0.24, 0.35) ††
Dimethylglycine	0.04 (0.02, 0.04)	0.07 (0.07, 0.08) ***	0.05 (0.05, 0.07) †
Sarcosine	0.14 (0.13, 0.15)	0.41 (0.32, 0.43) ***	0.26 (0.22, 0.33)
Creatine	1.12 (1.02, 1.23)	1.92 (1.85, 2.19) ***	1.58 (1.49, 1.70)
Guanidoacetate	0.23 (0.21, 0.28)	0.63 (0.60, 0.69) **	0.36 (0.35, 0.59)
Methylhydantoin	0.19 (0.17, 0.27)	0.62 (0.51, 0.66) ***	0.38 (0.30, 0.40) ††
Urea	11.19 (9.68, 13.58)	16.01 (15.45, 19.45) **	14.30 (13.01, 18.65)
Methionine	0.36 (0.23, 0.48)	2.32 (1.63, 2.94) ***	1.02 (0.78, 2.12)
Trigonelline	0.23 (0.21, 0.24)	0.36 (0.35, 0.41) ***	0.31 (0.30, 0.38)
Isoleucine	0.05 (0.05, 0.06)	0.13 (0.09, 0.25) **	0.06 (0.05, 0.09) †
Leucine	0.08 (0.06, 0.08)	0.30 (0.25, 0.63) ***	0.13 (0.11, 0.20) ††
Valine	0.04 (0.03, 0.04)	0.18 (0.13, 0.34) ***	0.07 (0.06, 0.13)
3-Methyl-2-oxovalerate	0.14 (0.12, 0.17)	0.22 (0.20, 0.28) **	0.15 (0.15, 0.18)
Taurine	8.79 (7.11, 9.93)	2.60 (2.21, 3.81) ***	7.36 (5.84, 8.64) †
Acetylcarnitine	0.01 (0.01, 0.02)	0.04 (0.04, 0.05) ***	0.02 (0.02, 0.02) †††
Isovaleroylglycine	0.26 (0.22, 0.28)	0.42 (0.34, 0.46) ***	0.32 (0.31, 0.33)
3-Hydroxy-3-methylglutarate	0.07 (0.06, 0.08)	0.11 (0.11, 0.13) ***	0.09 (0.08, 0.10) †
2-Methylglutarate	0.04 (0.04, 0.04)	0.07 (0.06, 0.07) ***	0.05 (0.05, 0.06)

The results are presented as the median (25th, 75th percentiles).

* $p < 0.05$, ** $p < 0.01$, and *** $p < 0.001$ for the difference between CM and DM.

† $p < 0.05$, †† $p < 0.01$, and ††† $p < 0.001$ for the difference between DM and LDM.

CM, control mice; DM, STZ-induced diabetic mice; LDM, losartan-treated diabetic mice.

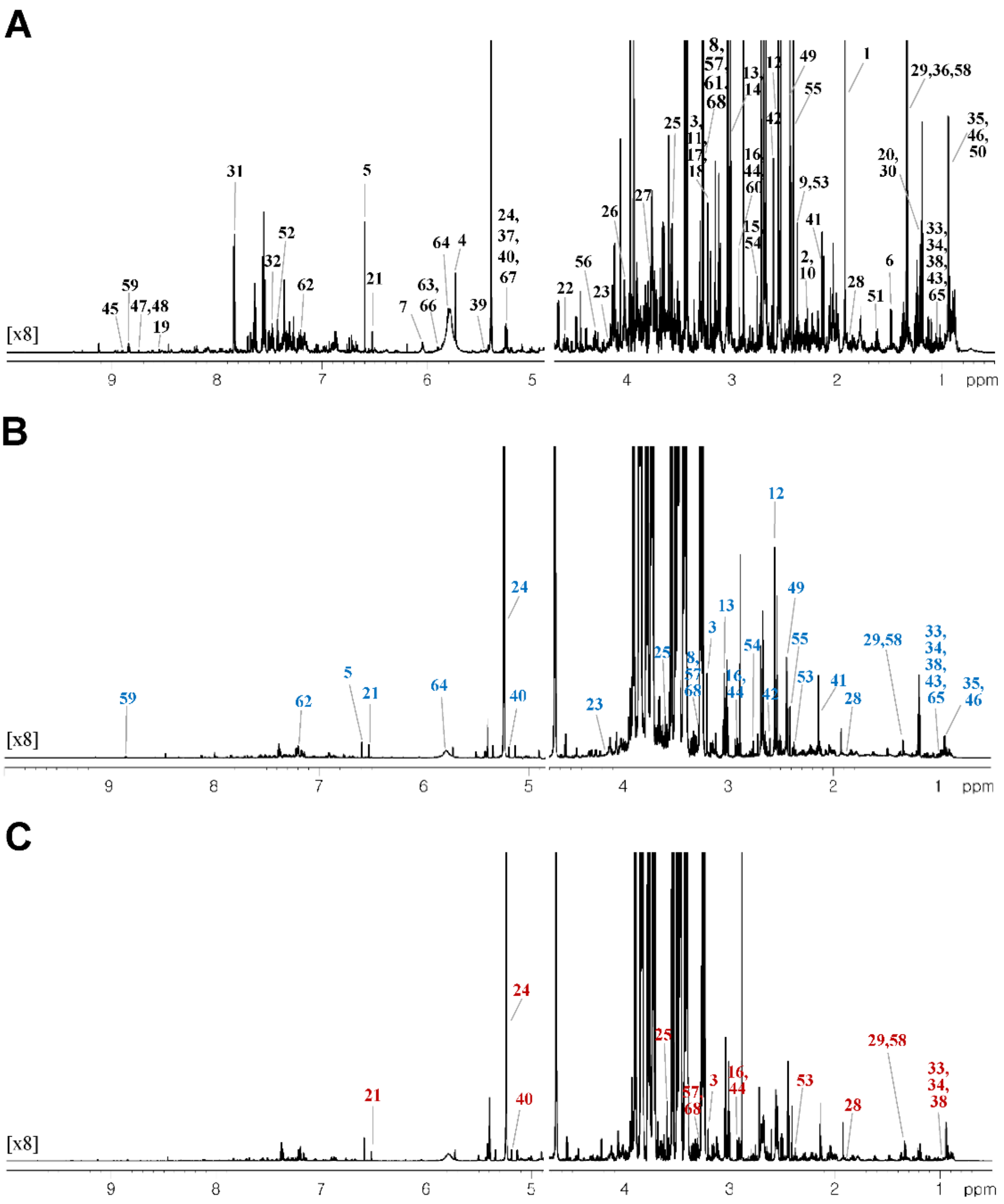

Figure S1. 1D ^{1}H NMR spectra of urine samples from CM (A), DM (B), and LDM (C). The identified metabolites are presented in black (68 metabolites), blue (significantly changed in DM) or red (significantly changed in LDM). 1, acetate; 2, acetoacetate; 3, acetylcarnitine; 4, cis-aconitate; 5, trans-aconitate; 6, alanine; 7, allantoin; 8, betaine; 9, N-carbamoyl-β-alanine; 10, carnitine; 11, choline; 12, citrate; 13, creatine; 14, creatinine; 15, dimethylamine; 16, dimethylglycine; 17, dimethyl sulfone; 18, ethanolamine; 19, formate; 20, fucose; 21, fumarate; 22, galactose; 23, glucarate; 24, glucose; 25, glycine; 26, glycolate; 27, guanidoacetate; 28, 2-hydroxyglutarate; 29, 3-hydroxy-3-methylglutarate; 30, 3-hydroxybutyrate; 31, hippurate; 32, 3-indoxylsulfate; 33, isobutyrate; 34, isoleucine; 35, isovaleroylglycine; 36, lactate; 37, lactose; 38, leucine; 39, maltose; 40, mannose; 41, methionine; 42, methylamine; 43, 2-methylglutarate; 44, methylhydantoin; 45, 1-methylnicotinamide; 46, 3-methyl-2-oxovalerate; 47, niacinamide; 48, nicotinamide N-oxide; 49, 2-oxoglutarate; 50, 2-oxoisocaproate; 51, 2-oxovalerate; 52, phenylacetylglycine; 53, pyruvate; 54, sarcosine; 55, succinate; 56, sucrose; 57, taurine; 58, threonine; 59, trigonelline; 60, trimethylamine; 61, trimethylamine N-oxide; 62, tyramine; 63, uracil; 64, urea; 65, valine; 66, xanthosine; 67, xylose; 68, myo-inositol.

1D ^{1}H NMR, one-dimensional proton nuclear magnetic resonance; CM, control mice; DM, STZ-induced diabetic mice; LDM, losartan-treated diabetic mice.

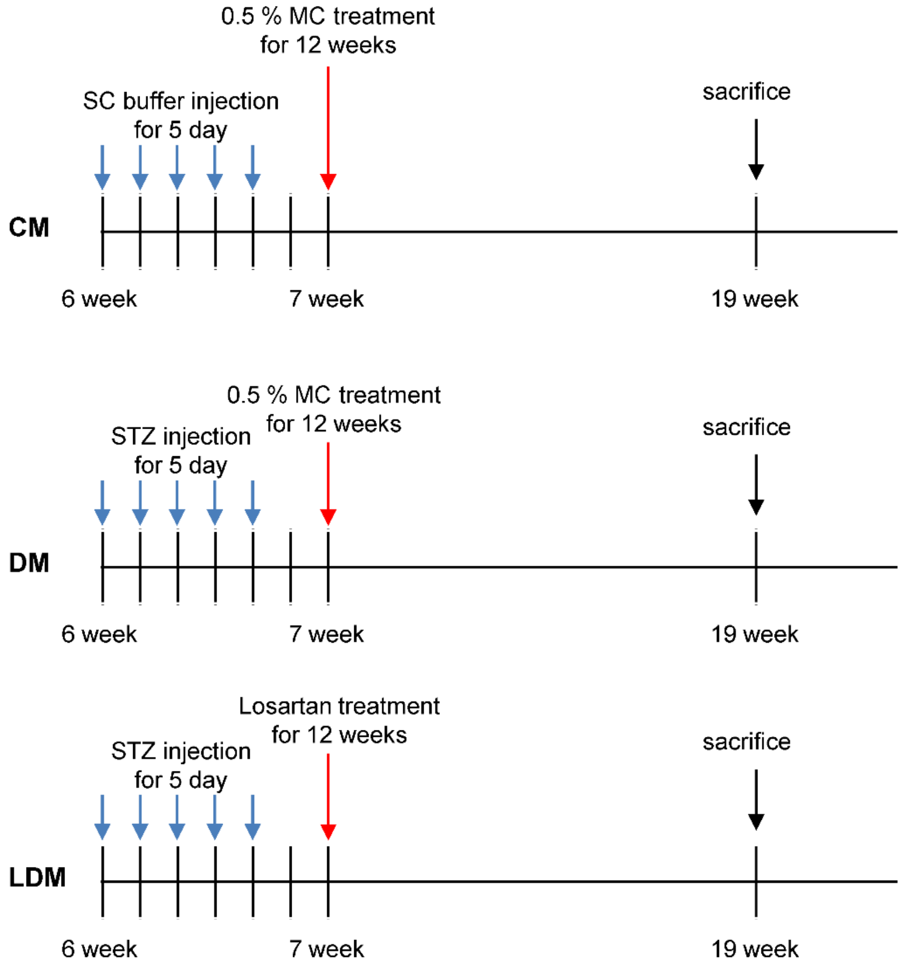

Figure S2. Experimental design. Design of the study for the STZ-induced diabetic mouse model and losartan treatment.

CM, control mice; DM, STZ-induced diabetic mice; LDM, losartan-treated diabetic mice; SC, sodium citrate; MC, methylcellulose.
